# Absorption of Insoluble Substances

**Published:** 1848-11

**Authors:** 


*Absorption of Insoluble Substances.*—Although numerous works have of late years been published on the physiology of absorption, this function is as yet far from being completely understood. The parts played by the veins and lymphatics respectively, is still but imperfectly known ; and the conditions necessary for the operation of absorption are still undetermined. Professor Oesterlen has made a discovery which, if verified, will completely subvert received theories. Having fed cats and rabbits five or six days consecutively, with food in which charcoal, minutely powdered, had been mixed, he found that on examining the blood under the microscope, particles of charcoal, identical with those which had been ingested, were abundantly visible. The blood was taken from the mesenteric and portal veins. The fluid of the thoracic duct did not contain any, neither did the urine or bile. Charcoal is not the only insoluble substance which is capable of being absorbed. Oesterlen found that Prussian blue can likewise be detected.—*Henle and Pfeufer Zeitschrift.*

